# The significance of a nineteenth century definition in the era of genomics: linitis plastica

**DOI:** 10.1186/s12957-017-1187-3

**Published:** 2017-07-05

**Authors:** Annamaria Agnes, Jeannelyn S. Estrella, Brian Badgwell

**Affiliations:** 1 Department of Surgical Oncology, The University of Texas MD Anderson Cancer Center, Unit 1484, 1515 Holcombe Blvd., Houston, TX 77030 USA; 2 Department of Pathology, The University of Texas MD Anderson Cancer Center, Houston, TX USA

**Keywords:** Gastric cancer, Linitis plastica, Borrmann type IV, Scirrhous carcinoma, Diffuse, Signet ring

## Abstract

**Background:**

Linitis plastica due to gastric adenocarcinoma is a condition with a long history, but still lacks a standardized definition and is commonly confused with Borrmann type IV, Lauren diffuse, and signet-cell type gastric cancer. The absence of a clear definition is a problem when investigating its biological characteristics and role as a possible independent factor for prognosis. Nevertheless, the biological behavior for linitis plastica, which is unique, may be valuable in risk stratification and have implications for treatment. A definition of linitis plastica based on molecular or genomic criteria could represent a useful starting point for investigating new targeted therapies.

**Main body:**

This literature review of linitis plastica will focus on the current classifications for gastric cancer, illustrating how the concept of linitis plastica relates to them in most cases and identifying a clear and reproducible definition. Moreover, the review will highlight the diagnostic challenges associated with linitis plastica, its prognostic implications, and the therapeutic options available. Future perspectives for its management are also addressed.

**Conclusion:**

Linitis plastica is a carcinoma with a scirrhous stroma, involving the submucosal and muscular layers of the stomach even in the absence of mucosal alteration. In most cases, the primary cancer cells are signet-ring cells or scattered cells in the context of a poorly differentiated carcinoma. Diagnosis is challenging. Staging should be thorough, including diagnostic laparoscopy in all cases due to the high incidence of peritoneal involvement. The prognostic significance of linitis plastica is still controversial. Curative-intent surgery, when feasible, should be performed, with a multimodality treatment approach. Cancer-stroma interactions are important features of this disease, and represent attaining potential target for future therapies. Future pathologic assessments of gastric cancer should report the stromal reaction in order to allow better characterization of the tumor.

## Background

Linitis plastica (LP) of the stomach is a long-known condition, with initial reports that date back to the sixteenth and seventeenth century [1]. It was defined as a distinct entity in 1859 by Dr. William Brinton, who described it as a benign disease with peculiar characteristics: the stomach was macroscopically thickened, with inconsistent evidence of mucosal ulceration; pathologically, it showed a prominent submucosal hypertrophy due to an increase in the connective tissue and prominent muscular hypertrophy. The choice of the term “linitis” was due to the presence of irregular bands of filamentous tissue in the hypertrophic submucosa, resembling fibers of linen. Clinically, this disease was unavoidably fatal without treatment [2]. Early reports on the presence of cancerous cells in the setting of LP were notable for the difficulty in identifying malignancy. Malignant cells, when detected, were often described as few and scattered [2, 3]. As a consequence, for many years, it was controversial if the condition was benign or malignant. Then, in 1953, Dr. Arthur Stout clarified the issue, proposing linitis plastica as a specific type of gastric carcinoma characterized by an excessive production of fibrous scar-like tissue, with areas in which only scattered cells were present. He also reported that the previous doubts on the malignant nature of LP were probably due to a failure in recognizing the presence of carcinoma cells by the past authors [4].

In the intervening years, multiple other classifications for gastric carcinoma have been established, reflecting the heterogeneity of this malignancy [5–8]. Each is based on different macroscopic and microscopic aspects of the tumor. LP had been associated with gastric carcinoma, but Stout’s classification did not take hold, and LP was never included in any of the other staging systems. In the following years, the definition of LP was separated from the presence of fibrous tissue, becoming more generalized and being increasingly associated with diffuse carcinomas with infiltration of the gastric wall, resulting in the stomach having a stiffened appearance and a partial or complete lack of distensibility [9]; occasionally, the term has also been extended to include other conditions associated with thickening of the stomach wall without any fibrous component at all (i.e., lymphoma) [10, 11].

Recent reports of LP lack a clear and standardized codification. “Linitis plastica” is used interchangeably with “Borrmann type IV carcinoma,” “scirrhous carcinoma,” “signet-ring cell carcinoma,” and “Lauren diffuse carcinoma” [11, 12]. However, it is not clear if these terms correctly define this condition, as only some of the tumors in each of these categories have the features of LP.

Due to the lack of agreement on the clinical significance of LP and the difficulty in attributing this condition to the common classification systems, some authors have proposed to abandon this definition [13]. Others, nonetheless, still recognize in LP a specific type of gastric cancer with a distinct growth pattern and biological behavior, and advocate that the identification of such a subset of gastric cancer patients could be useful in risk stratification, in identifying a target for therapeutic management, and in guiding future research [14–18].

In recent years, the role of LP has come under discussion again with a focus on its prognostic significance. Many authors have associated this condition with diminished survival compared to non-linitis tumors, even proposing that this disease should be considered non-surgical [14, 16, 19]. Conversely, there are reports noting a similar prognosis between LP and non-LP patients after stage-stratification or application of other adjustment methods [17]. Even in these studies that focused on investigating LP, the definition of LP is not uniform; hence, it is very difficult to interpret their results.

In this review, we assess the significance of LP in relation to the current classification systems, and propose working towards a univocal definition useful for clinical and research purposes. Furthermore, we summarize the diagnostic challenges of this condition, its prognostic aspects and its therapeutic implications, and discuss future research and clinical trial opportunities.

## Main text

### Current classifications for gastric carcinoma and their relations to LP

Gastric cancer (GC) has several classifications related to its macroscopic and microscopic aspects. All are commonly used, but none of them has been accepted as the standard system. We hereby review the most common ones, addressing their overlap with LP.

**Fig. 1:**
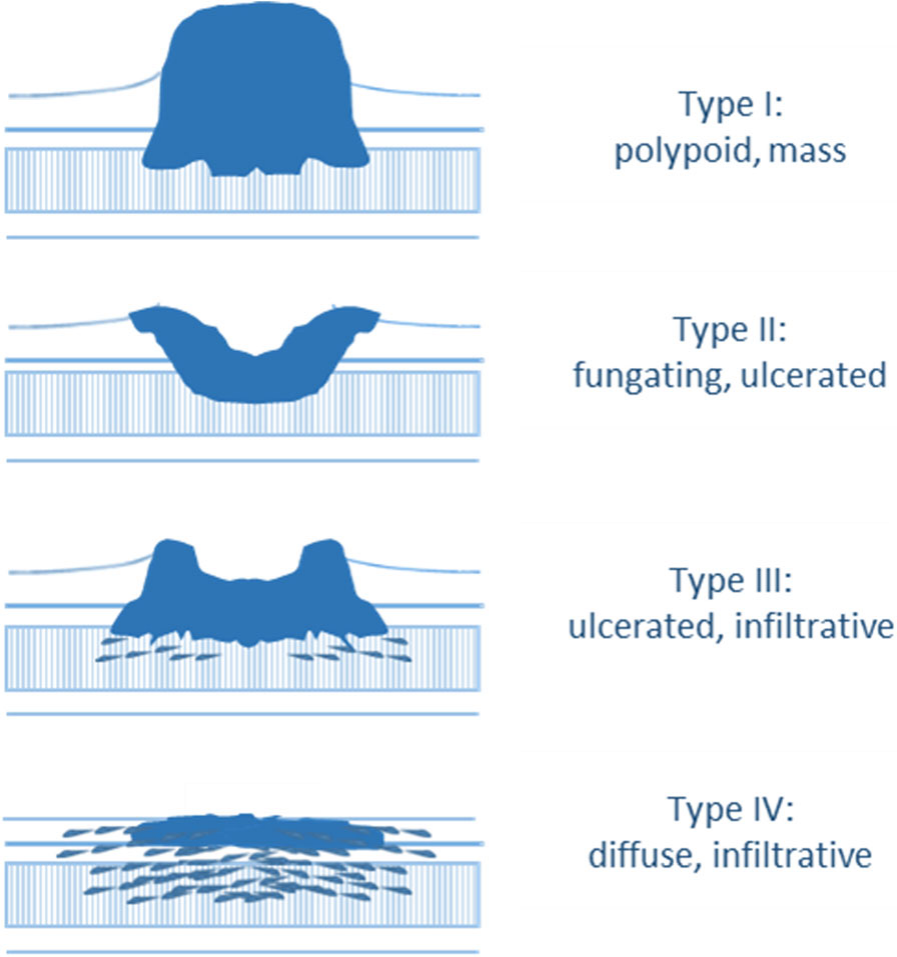
Borrmann types

Borrmann classification: first proposed by Borrmann in 1926 [5], this classification is most commonly used in Eastern countries [8, 12]. It is based on the macroscopic endoscopical/endoluminal aspect of the tumor and is most useful as a preoperative assessment tool and a prognostic factor. Borrmann type IV tumors are described as diffuse and infiltrative (“tumor without diffuse ulceration or raised margin, the gastric wall is thickened and indurated, and the margin is unclear”) [8] (Fig. 1). They represent 8–17% of GCs [20]. Clinicopathological characteristics of Borrmann IV tumors include an association with younger age and female gender, detection in an advanced stage, high incidence in the middle third or diffusely involving the entire stomach, predominance of Lauren diffuse-type and undifferentiated histology, deeper invasion of the gastric wall, a high rate of peritoneal and lymphatic involvement, low rate of liver metastases, and a high rate of recurrence after curative-intent surgery [20–22]. The natural history of Borrmann IV tumors has been investigated, and reports state that the precursor of a Borrmann IV tumor is probably a Borrmann type 0-IIc lesion (a superficially depressed early GC) and not a Borrmann III lesion [11, 23–25]. Borrmann type III tumors, instead, represent 59–68% [21, 22, 26] of GCs. They are characterized by both an infiltrative and ulcerative pattern [8] (Fig. 1), and their clinicopathological characteristic partially resemble those of Borrmann IV tumors [26]. Both Borrmann III and Borrmann IV tumors show an infiltrative pattern, and even Borrmann III tumors may show a consistent desmoplastic reaction [12]. At the same time, not every Borrmann IV tumor presents with the typical demoplastic characteristics of LP [11]. Therefore, LP is often improperly defined as a Borrmann IV tumor.

Cancer stromal volume classification: this microscopic classification is part of the Japanese classification [8]. It includes cancers with a medullary type (scanty stroma), a scirrhous type (abundant stroma), and an intermediate type. A similar system is utilized in the World Health Organization (WHO) classification (described below) [7], although tumor stroma is less commonly categorized by Western pathologists. In the Eastern setting, this classification is more commonly applied; however, there are few studies focusing on the clinicopathological aspects of tumors identified by the scirrhous classification system. Scirrhous cancers, in accordance with the original definitions of LP [2, 4], are strictly related to the LP phenotype, which presents in its classical form only when the submucosa is diffusely fibromatous [18]. In LP, the scirrhous component spreads primarily through the submucosa, with so much tropism that in some cases of LP the only endoscopic finding is an increase in the folds of the stomach [2, 27]. Scirrhous carcinomas may be a Borrmann type III or IV [12, 28], and is reported to represent 5–43% of GCs [12, 16, 28, 29]. Clinicopathological characteristics of these tumors, in comparison with medullary carcinomas, include younger age, female prominence, more advanced stage, deeper invasion in the gastric wall, poor histologic differentiation, more peritoneal diffusion, less hematogenous diffusion, and more lymphatic invasion [16, 28].

Lauren classification: this microscopic classification divides gastric tumors into diffuse, intestinal, or mixed and indeterminate types. Diffuse adenocarcinomas are defined by their growth pattern as tumors infiltrating the stroma as discohesive tumor cells arranged singly and in small clusters. The intestinal type is defined by its cytoarchitecture, and characterized by cohesive cells which form gland-like structures. Mixed tumors have both an intestinal and diffuse component, while indeterminate types include most of the undifferentiated tumors [6, 7, 30]. Diffuse tumors account for 32–49% of GCs [31, 32]. There is a significant correlation between the diffuse histotype, the Borrmann III and IV types, and the scirrhous stromal category [31]. Diffuse histology has been frequently linked to LP [33], and it is also the typical pattern of familial hereditary diffuse GC, which in its advanced stages often presents as LP [7]. Clinicopathological characteristics of diffuse tumors include younger age, higher rate of incidence in the female gender, more advanced stage at presentation, more poorly differentiated tumors, more lymphovascular invasion, and more peritoneal dissemination [31, 34]. Unfortunately, despite its clinical success, the Lauren classification is broad, and may not valorize all the tumor features. In addition, mixed tumors have been reported to have a specific impact on prognosis, and to have possible subcategories themselves [35], and adding to the complexity of this classification is that mixed and indeterminate tumors have often been classified in either the intestinal or the diffuse category for reasons of simplicity.

WHO classification: The WHO classification is a descriptive system which defines five main types of gastric carcinoma: tubular, papillary, mucinous, poorly cohesive (including signet-ring cell carcinomas and other variants), and mixed adenocarcinomas. Lauren diffuse carcinomas most often have a poorly cohesive histotype. Signet-ring cell (SRC) carcinomas are defined as tumors composed of cells containing intracytoplasmic mucin and eccentrically placed nucleus, in a proportion >50%. They may form lace-like glands or a microtrabecular pattern in the mucosa, or extend to deeper layers with significant desmoplastic reaction. Irrespective of the category, SRCs may also be present in different tumors, as poorly cohesive variants, mucinous tumors (defined by extracellular mucin >50%), and mixed carcinomas (defined by a clonal mixture of both glandular and poorly cohesive aspects) [7]. Hereditary diffuse GC typically presents as a diffuse gastric carcinoma containing SRCs, and often with features of linitis plastica [7, 33].

The WHO classification also describes four stromal reactions (desmopasia/scirrhous reaction, lymphocytic infiltration, stromal eosinophilia, and a granulomatous response) and presents a grading (well, moderate, and poorly differentiated), which should be applied only to tubular and papillary variants of GC [7].

The WHO classification represent an exhaustive depiction of the various possible type of tumors by microscopic pattern, but has the limitation of being exclusively descriptive, not including the histogenesis of gastric adenocarcinoma, nor its biological behavior. This limitation is problematic when considering the WHO categories as prognostic factors, and the strict classification of SRC carcinoma (SRC >50%) does not account for the possible clinical significance of the presence of any SRCs, or for the possible significance of mucinous SRC tumors. Indeed, the mucinous category may contain SRCs in mucin pools, and mixed carcinomas are reported to have a more detrimental prognosis in regards to the presence of a SRC component [7]. In addition, it has been advocated that a subgroup of less differentiated mucinous tumors may have a pattern and prognosis similar to those of signet-ring cell tumors [36]. In general, however, SRC tumors account for 16–32% of all GCs [13, 37] and present similar clinicopathological characteristics to Borrmann IV, scirrhous, undifferentiated, and diffuse GC types [37]. Poorer prognosis has been reported by some authors [13] in comparison with other histotypes, while others have demonstrated equivalence of the prognosis in early stages [38] and in advanced stages after stage-adjustment [39].

Due to the variabilities in their diagnostic criteria and to the frequent coexistence of different histologic features in the same tumor, current microscopic classifications are not completely reliable. The comparison between preoperative biopsy and resection specimens shows disagreement in 25–35% of the cases with regards to Lauren classification and 16% with regards to the WHO system [30, 40]. Inter-observer disagreement on resected specimens ranges from 17 to 32% for the Lauren classification and from 21 to 32% for the WHO classification [30]. More confusion is induced by the fact that these classifications are used regionally (the application of the Lauren classification is not common in the East, while the Borrmann and stromal classifications are rarely used in the Western setting). For all these reasons, a manageable, reproducible, and universal classification system would be helpful.

Various novel classification systems, based on the genomic and epigenetic features of the tumors, are currently being developed for GC [41–44]. They have several theoretical advantages, being reproducible without room for subjective interpretation, abolishing categories as “indeterminate” or “mixed type” [33], and being directly linked to the biological behavior of the tumor. Furthermore, they could allow for the conception and development of targeted therapies [45]. Nonetheless, these methods are still expensive and not easily applicable [43, 45, 46], and univocal classifications have yet to be developed. One current limit is represented by the low-frequency of certain molecular alterations, and the consequent need for large-scale testing [47]. In regards to GC, the categories identified thus far are still heterogeneous, and there are still a vast number of potential targets for testing. None of the classifications proposed are specifically linked to scirrhous tumors or LP, but it seems highly probable that the LP phenotype may reside in the G-DIF (64% concordance with Lauren diffuse tumors) [41], mesenchymal (high activity of epithelial-to-mesenchymal pathway, TGF-β) [44], GS (more Lauren diffuse tumors, defects in cell adhesion) [42], and MSS/EMT (Lauren diffuse, epithelial-to-mesenchymal transition, less liver metastases) [43] subtypes defined by those classifications.

### Actual significance and features of linitis plastica

Linitis plastica of the stomach is a distinct phenotype of gastric tumors. This term, as originally defined, includes both microscopic and macroscopic features.

#### Histology

The most characteristic feature of LP is the macroscopic thickening of the stomach wall, often diffusely involving the entire stomach, which has been described in detail since its early reports as an impressive increase in the submucosal connective tissue in the form of immature and mature stroma [2, 3] with hypertrophy of the muscle layer and subserosal thickening [48]. These characteristics strictly resemble those of scirrhous carcinoma, which, as mentioned above, is a particular form of GC in which cancer cells trigger a stromal reaction involving mature and immature fibrosis (which are characterized, respectively, by the presence of collagen I and III) [49, 50]. The scirrhous reaction is almost always triggered by poorly cohesive neoplastic cells, often with signet-ring morphology. More rarely, cases of schirrous tumors accompanied by moderately differentiated adenocarcinomas have been reported [13, 15–19, 28].

Every cancer is composed by both the cancerous cells and their environment that consists of endothelial cells, inflammatory cells, and connective tissue (matrix and fibroblasts) [51, 52]. The importance of these interactions is increasingly being recognized, in contrast to previous research which has exclusively focused on cancer cells. Scirrhous tumors are characterized by a complex interaction between cancer cells and cancer-associated fibroblasts (CAFs), which may represent up to 90% of the tumor and appear to have a primary role in cancer progression. The origin of CAFs is under investigation; these cells seem to be heterogeneous, as they may be local fibroblasts, cells recruited from the bone marrow, or pericytes which undergo endothelial to mesenchymal transition [49, 52, 53]. Cell-stroma interactions have been associated to activated TGF-B, HGF (c-met ligand), FGF7, and other soluble factors [53, 54], yet they are complex and still have to be clarified.

**Fig. 2:**
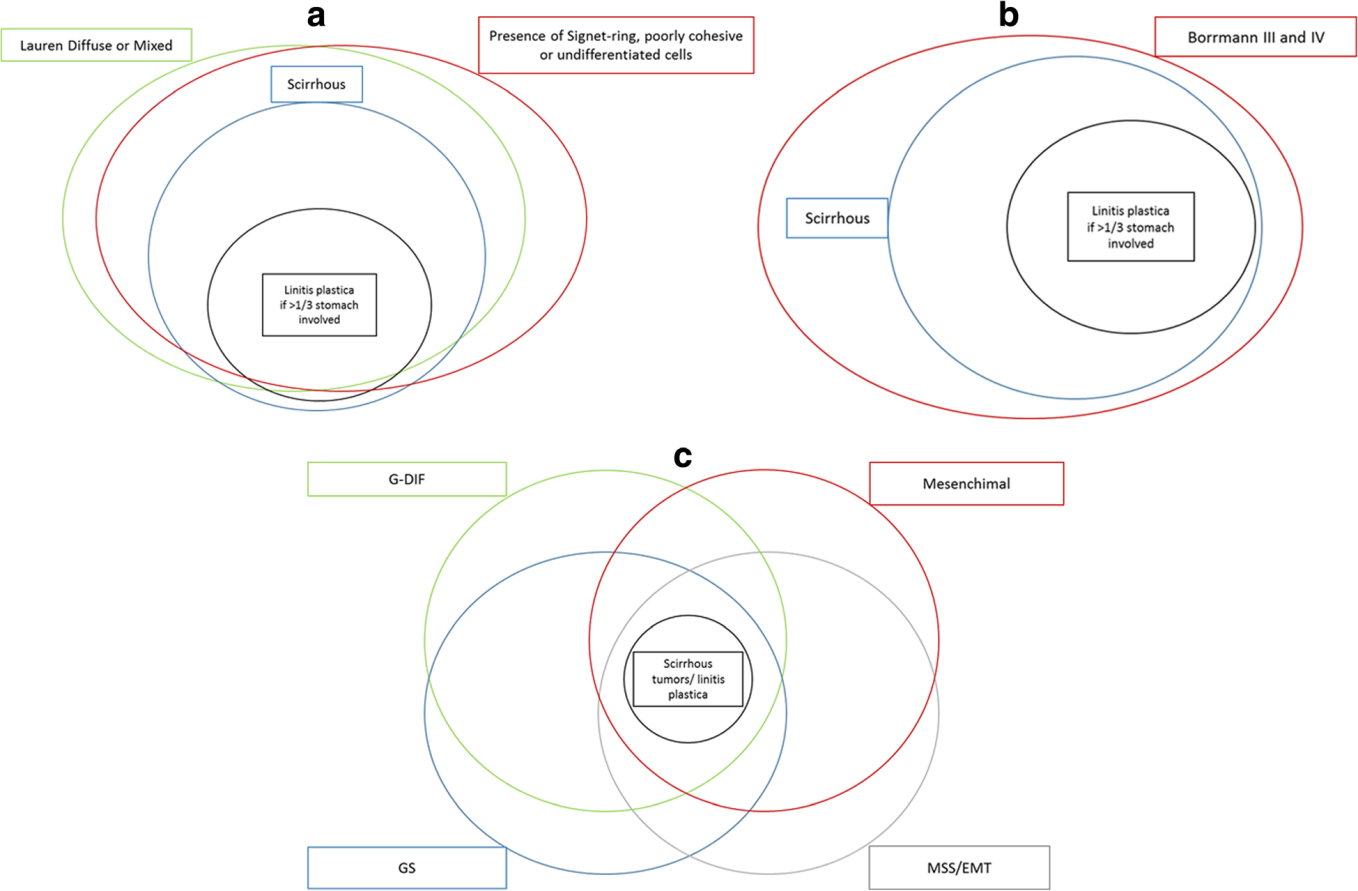
Venn diagrams depicting the relations between the current classifications and linitis plastica. **a** Western setting. **b** Eastern setting. **c** Molecular classifications

Cells should not be analyzed without a parallel analysis of their environment, and in every case of properly defined scirrhous tumors, signet-ring or other poorly differentiated cells should be documented in conjunction with a scirrhous stroma (Fig. 2). Cases of scirrhous stromal reaction triggered by moderately differentiated intestinal tumors without the above-mentioned cells have been documented as well, but seem to be extremely rare (Table 1).

**Table 1:** Characteristics of differing classifications of linitis plastica in the literature

Author, year	Country	Diagnostic criteria	Age	Gender % (M/F)	Histology
Aranha, 1989 [76]	USA	Scirrhous + extension (unclear)	62 (42–80)	42/58	100% poorly differentiated or anaplastic carcinoma, with or without signet ring cells
Hamy, 1999 [77]	France	Histology (infiltrating, SRCs)	63.4 ± 25.6	59/41	100% SRCs, 77% scirrhous
Kodera, 2004 [70]	Japan	Histology (scirrhous)	56.5 ± 11.6	53/47	Scirrhous
Kodera, 2008 [75]	Japan	Barium meal or endoscopy	59 ± 11.5	51/49	–
Schauer, 2011 [15]	Germany	Locally advanced + SRCs	57.7 (28–83)	1:1	Diffuse
Endo, 2012 [16]	Japan	Scirrhous + extension >2/3	69 ± 7.7	58/42	16% SRC carcinoma, 63% poorly differentiated, 21% moderately differentiated
Pedrazzani, 2012 [14]	Italy	Lauren diffuse + thickening of the gastric wall >1/3	68 (29–89)	56/44	Diffuse
Jafferbhoy, 2013 [19]	UK	Nonspecified	75 (59–87)	–	–
Blackham, 2016 [17]	USA	Endoscopic assessment or intraoperative assessment or histologic evaluation (Borrnann, scirrhous)	61.1 ± 13	47/53	98% poorly differentiated 2% moderately differentiated
Thompson, 2016 [78]	UK	Endoscopic or radiologic features (unclear)	69.6 ± 13.6	50/50	Diffuse

#### Secondary linitis plastica

Insights on the biological behavior of scirrhous and LP tumors may also be gathered from the features of secondary LP. Secondary LP features may be found in other hollow viscus or cystic organs, such as the bowel, bladder, and ovaries [27, 55]. Frequently, SRCs are detected in these types of secondary lesions [18, 56]. At the same time, even secondary LP of the stomach has been described. The most commonly reported cases of secondary LP are those associated with metastatic invasive lobular carcinoma of the breast, which presents with identical radiologic [9] and nearly identical histologic characteristics to primary linitis plastica [57]. This condition may arise even 10 to 15 years after the initial diagnosis of breast cancer, rendering the past medical history essential to consider this diagnosis. Notably, lobular breast carcinoma is also a feature of hereditary diffuse GC [33], and it often contains scattered SRCs [57]. Scirrhous GCs seem to be more frequent in the female gender than other stromal types [16, 28], and have been reported to express ER in diverse studies [57]; these features could be imputed to gastric CAFs being a common soil for tumors in which hormones have a specific role. On the other hand, some authors have suggested that, if a high level of suspicion was kept and routine testing for biomarkers of breast carcinoma applied, a secondary breast origin for LP would be detected in more cases [57, 58].

#### Mucosal involvement

**Fig. 3:**
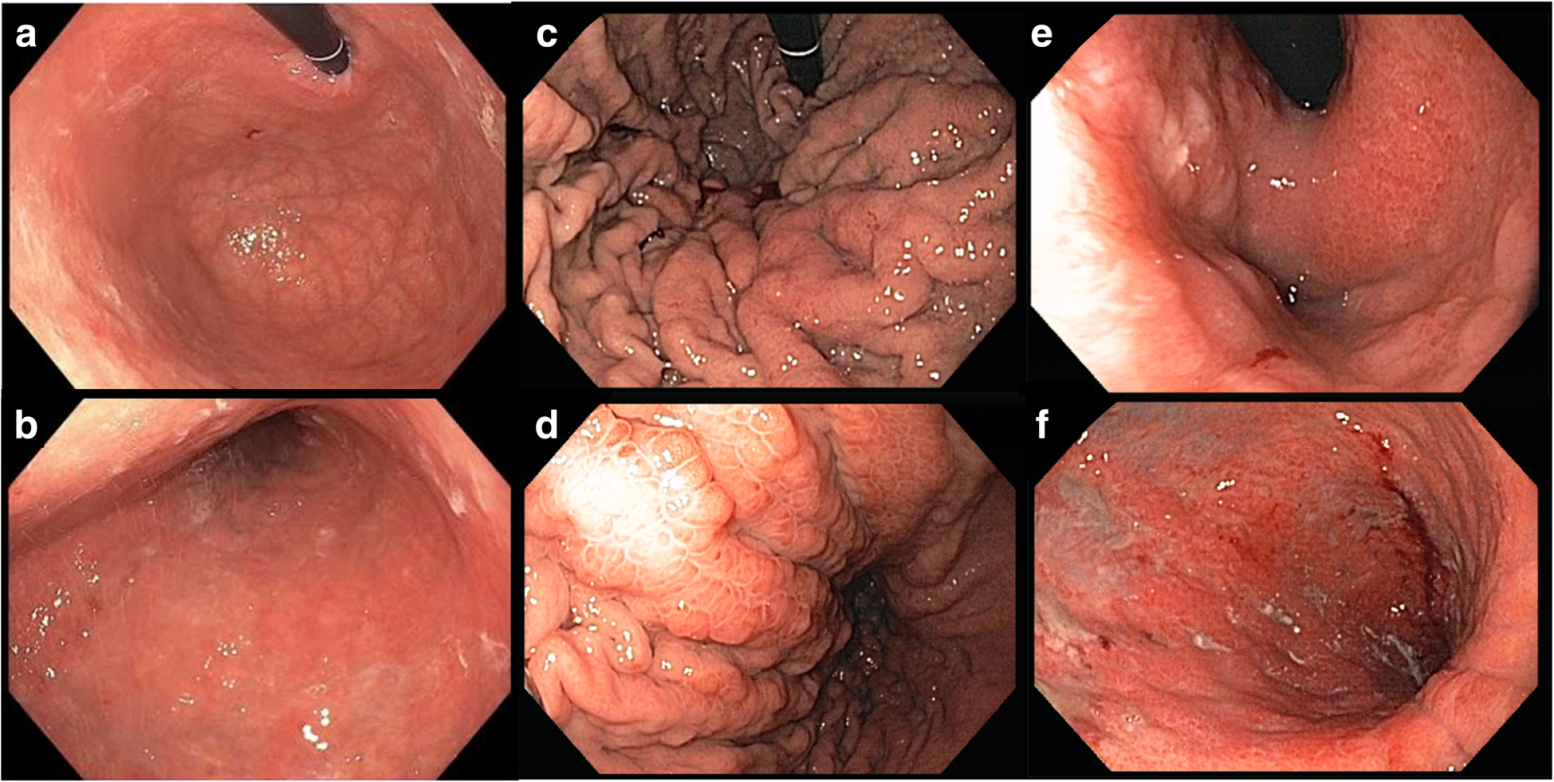
Endoscopic aspect of two different types of linitis plastica: the waffle-like (**c**, **d**) and the flat type (**e**, **f**). **a, b** The aspect of a normal stomach

In regards to the quality of the mucosal involvement, two different subtypes of linitis plastica have been described (Fig. 3). In the first type, *giant-fold* or *waffle-like*, the mucosa demonstrates a characteristic morphological change consisting of an enhancement of the design of the folds, which remain flexible but appear prominent and crossing one another. This effect may be due to the relatively normal state of the mucosa in comparison to the involvement and contraction of the submucosal and muscular layers [2, 11]. The pattern of waffle-like LP has been extensively described in several Japanese studies, and in case reports of patients refusing surgery and subsequently being followed for years [11, 23–25]. The first lesion generally originates from the proximal or middle stomach, near the great curve, as a type IIc (flat depressed) early GC. This condition may remain stable for 2–5 years (*slow-phase*), until the lesion progresses to advanced GC and ulcerates reaching the submucosal layer. At this point, the ulcerative lesion can persist or heal, while the submucosal involvement, once a scirrhous reaction is initiated, enters a *fast-phase*, involving the entire stomach (LP) in about 1 year [23–25].

In the second type, the *flat* type, submucosal involvement is paralleled by mucosal thickening or atrophy [11]. This type most commonly originates from the antrum, near the lesser curvature, and then extends to involve the antrum circumferentially. Flat type LP development has not been extensively studied. The difference between the characteristic mucosal pattern in waffle-like LP and the mucosal flattening and induration in flat type LP may due to diffusion of the neoplasm in a more superficial plane (involving the lamina propria, the muscolaris propria, or the mucosa itself) in flat type. The tumor is thought to originate near the pylorus, involve the antrum circumferentially, and extend to the entire stomach [9, 19, 27]. The flat type is commonly believed to be the most common in Western settings [11]. However, even if the original description by Brinton, most of the LP cases were identified as originating from the distal stomach, with hypertrophic mucosal folds [2], and a mixture of both subtypes [11]. In 1990, a US study demonstrated that up to 88% patients with scirrhous tumors present with the radiological features of thickened gastric folds. Moreover, in this study, 38% presented with mainly proximal involvement and 35% with mainly distal involvement [9]. Conversely, a 2004 Korean survey has reported a 22% rate of primarily proximal and a 59% rate of primarily distal involvement [12]. Therefore, the distinction between these two types is not clear-cut.

#### Extension and macroscopic features

LP does not always present as complete involvement of the stomach. It may appear in plaques which gives the appearance of a segmental lack of distensibility [9, 12]. Localized and diffuse forms of linitis were found in the early reports of this condition [2, 3], and recent evidence seem to have confirmed that the submucosal and muscular involvement by the scirrhous reaction is progressive. Indeed, Endo et al. [16] have been investigating localized schirrous tumors versus extended forms of LP, identifying similar gender rates, histology, and lymphatic invasion between these tumors, and a progression from younger towards older age and from less towards more advanced stage between the localized and diffuse forms. In relation to the Japanese studies on the growth pattern of scirrhous tumors [24, 25], many have proposed that the localized form may correspond to a relatively stable scirrhous disease, which is followed by a rapid phase of growth in which the desmoplastic reaction rapidly involves most of the stomach [16].

There are also multiple proposed definitions for LP based on extent of gastric involvement (Table 1). Nakamura et al. [25] defined typical LP as involvement of more than ¼ of the stomach; Pedrazzani et al. [14] proposed a cut-off of 1/3, and Endo et al. [16] a limit of 2/3; each of these classifications has only been applied once, and neither of them specify if the involvement had been pre- or postoperative or if it is intended for both the anterior and the posterior aspect of the stomach.

#### Definition: postoperative or preoperative?

**Fig. 4:**
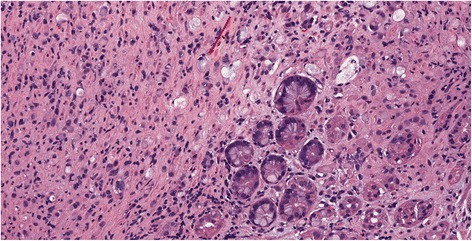
Histological features of scirrhous tumors. Note the prominence of the stroma (especially on the *left*). *Red arrow*: a scattered signet-ring cell

LP is, by the original definition, a scirrhous tumor that spreads through the submucosal and muscular layers of the stomach, with thickening of its wall and loss of distensibility. The presence of poorly differentiated, poorly cohesive, or SRCs is often involved (Fig. 4).

However, the original definition is based on autopsies and surgical specimens, and it should be noted that it may be difficult to obtain a reliable biopsy documenting both the predominance of the stroma and the cancerous cells in the preoperative setting. Moreover, many of these patients, affected by advanced disease, would not undergo gastrectomy; therefore, analysis on postoperative surgical specimens would not always be possible. In addition, the increasingly common practice of administering preoperative therapy in the form of systemic chemotherapy or radiotherapy may hamper the identification of the typical stromal reaction of LP, as fibrosis is often a consequence of preoperative therapy [59].

**Fig. 5:**
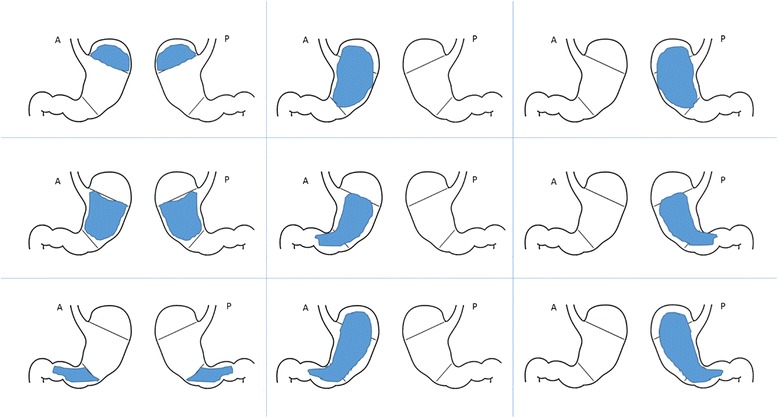
Different minimum exensions of the scirrhous reaction which may define a linitis plastica tumor (1/3 of the stomach surface)

Therefore, on the one hand, the identification of the specific morphologic characteristics of a tumor with a special biological behavior remains extremely important to improve understanding of the disease (see next paragraph), on the other there is the need for a simpler definition (univocal as well), which could be used in the clinical practice to aid oncologists and surgeons to stratify the prognosis of gastric cancer patients and define the therapeutic strategy even preoperatively. Thus, the most useful definition would probably be a preoperative one. To be simple and easily obtainable, it should also be macroscopic, with a cut-off extension over which the LP phenotype is clearly identifiable. This would guarantee a uniform identification of the condition and represent a good surrogate for the tumor phenotype. In our opinion, this definition could include thickening of the gastric wall, with lack of distensibility, which involves more than 1/3 of the gastric surface, both as a circumferential involvement of more than one area, or a semicircular involvement of more than two areas (Fig. 5).

#### Perspectives

What could be the role of distinguishing LP in the era of genomics? The concept of linitis plastica is unique in that it links diverse aspects of specific subtypes of tumors (macroscopic, microscopic, and environmental). Maintaining the LP definition and linking it to the stromal reaction could be useful for future studies and for genomic investigations, as patients with typical LP may be a relatively homogeneous subset of patients with a distinct tumor biologic behavior, in which specific genetic and epigenetic changes may be easier to detect than in the entire class of G-DIF and GS tumors. In particular, interesting expression changes are those happening in the cancer cells when the tumor activates the scirrhous response and the epithelial-to mesenchymal transition [43]. For years, oncological researchers have been focusing on cancer cell analysis, while more recently the attention is shifting towards analysis of the cell-environment relation [53], of which scirrhous tumors and linitis plastica seem to be a perfect example. In this regard, direct molecular profiling of LP CAFs would also be of great interest.

### Linitis plastica: diagnostic challenges

**Fig. 6:**
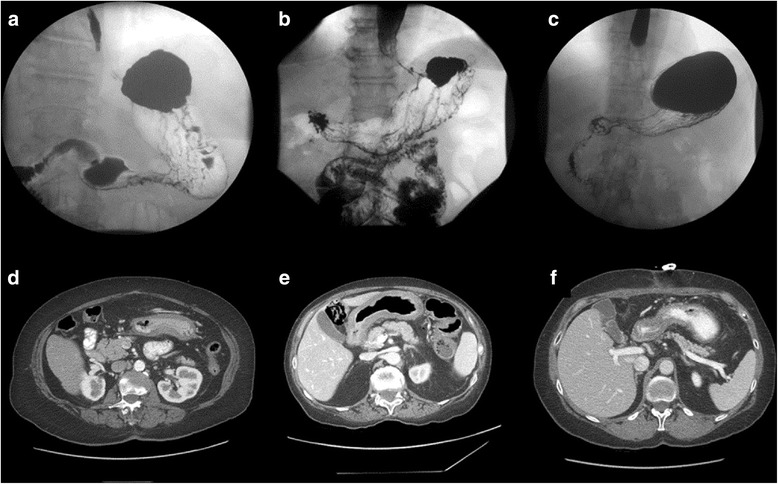
Upper gastrointestinal imaging (**a**–**c**) and computed tomography (**d**–**f**) diagnostic features of linitis plastica in three different patients (patient 1: **a**, **d**; patient 2: **b**, **e**; patient 3: **c**, **f**)

Typical symptoms of LP are dyspepsia, nausea, vomiting, and anorexia. Unfortunately, those symptoms are not reliable for establishing a timely diagnosis, as they usually present insidiously, and manifest only in an advanced stage. Available diagnostic instruments for this condition include endoscopy, endoscopic ultrasound (EUS), upper gastrointestinal contrast studies (UGI), computed tomography (CT) and 18-fludeoxyglucose (18-FDG) positron emission tomography (PET) scans, and magnetic resonance imaging (MRI) (Fig. 6).

Endoscopy is considered the gold standard for the diagnosis of GC. However, the peculiar spread pattern of LP tumors involves primarily the submucosa and muscularis propria of the stomach, while mucosal involvement is inconstant and may present as nonspecific gastritis or normal mucosa in up to 30% of cases [11]. The flat type could be confused with atrophic gastritis and suspected by endoscopy sometimes only due to the lack of distension of the stomach wall. Waffle-like appearance of the mucosa is more characteristic, even if biopsies have the same low diagnostic yield. Due to their poorly cohesive nature, cancer cells are often scattered between the tumor stroma [12, 56] (Fig. 4) or even absent in some sections [27]. Indeed, studies show high rates of non-diagnostic biopsies (30–36%) [9, 60]. Sometimes, the delay in the histologic diagnosis may represent a serious challenge, especially when the clinical and instrumental suspicion of LP is strong, as several different diagnoses are possible in the presence of hypertrophic mucosal folds and/or scarce distensibility of the stomach (gastric lymphoma, Ménétrier disease, granulomatous diseases and metastasis) and not all of them are surgical [9, 11, 27]. Gastrectomy is a major procedure with considerable morbidity and mortality, and many clinicians would not perform resection until cancer has been proven by biopsy.

Several endoscopic strategies to better diagnose this disease have been proposed. EUS features include submucosal and muscular thickening, and EUS fine-needle aspiration allows reaching of the submucosal layer [11, 18]. Even with this strategy, however, negative biopsies have been reported [61]. For this reason, EUS is also not considered the gold standard in diagnosis of LP. Endoscopic mucosal resection, due to the deep involvement of the stomach wall and the greater submucosal involvement, may not be ideal [62]; instead, other authors have been proposing a mucosal flap with submucosal endoscopic resection [61]. In addition, after the introduction of new-generation endoscopic techniques (endocytoscopy and endomicroscopy), there have been various reports on the detection of SRCs in vivo [63, 64].

Barium studies can be a useful diagnostic instrument, as they could document the thickening of the mucosal folds, and assess in real time the segmental or complete lack of distensibility of the gastric wall. In 2004, Park et al. reported UGI to be more reliable than endoscopy in detecting both the Borrmann type and the location of the primary tumor when evaluating scirrhous carcinomas [12]. UGI has been progressively discarded as a diagnostic technique for gastric cancer diagnosis and staging; nevertheless, given the low sensibility of conventional endoscopy, it remains of valuable support in evaluating this condition.

CT allows for comprehensive staging of the tumor, and could give rise to reasonable suspicion when identifying a stomach with thickened walls, which presents with complete flattening of the mucosal folds or thickened folds even after distension [55, 65] (Fig. 6). Two studies focusing on the diagnostic yield of CT have described a specific enhancement pattern in LP patients [55, 65]. 18-FDG PET, by the contrary, may have scarce diagnostic significance, as poorly differentiated, diffuse, mucinous, and SRC carcinomas have all been reported to be low in 18-FDG uptake [66].

MRI has been recently proposed as an alternative to CT, due to its advantages in characterizing tissue nature and obtaining soft tissue contrast [67], but the topic is still controversial [68]. One recent study identified a significant association between the apparent diffusion coefficient obtained with diffusion weighted imaging during MRI and different histological types, degrees of differentiation, and Lauren classification [69].

Suspicion of linitis plastica should prompt consideration for laparoscopy in staging. In consideration of the well-known peritoneal tropism of the disease, a diagnostic laparoscopy with peritoneal washings should be mandatory to complete the staging, if technically possible and not limited by previous surgery. Indeed, previous studies had reported positive cytology and macroscopic carcinomatosis in 49–64% of patients with LP undergoing diagnostic laparoscopy [15, 70, 71]. Moreover, laparoscopic biopsies of the gastric wall could be diriment in selected cases (i.e., when a histologic diagnosis is not obtainable even after repeated endoscopies).

A gold-standard diagnostic instrument for LP has yet to be defined. Proposals for diagnostic algorithms have been reported [11]. However, in the absence of clear definition for LP, the development of a diagnostic strategy is difficult. Moreover, macroscopic and microscopic assessments of the stomach are both required to identify the condition. If the suspicion is strong, even in the absence of a positive biopsy, the possible diagnoses should be discussed with the patient, and a diagnostic laparoscopy proposed to avoid deleterious diagnostic delays.

Future diagnostic advancements may be obtained by the use of blood-based biomarkers. In 2000, Ichikawa et al. proposed a high level of trypsinogen as a simple and specific marker to diagnose linitis plastica, but this marker has not been further tested or introduced in clinical practice [72]. “Liquid biopsy” of circulating tumor cells, cDNA, or miRNA may represent a future perspective [73, 74], especially as genomic and epigenetic characteristics of GC are better understood.

### Linitis plastica: impact on prognosis

Diverse studies have evaluated the prognostic significance of LP throughout the years (Table 2) [14–17, 19, 70, 75–78]. The absence of assessment of the stromal component and the use of non-standardized definitions (based on heterogeneous microscopic and macroscopic criteria) represent a notable limit of these studies. Nonetheless, some common prognostic features have been detected.

**Table 2:** Prognostic implications of linitis plastica in the literature

Author, year	Study design	LP sample size	Stage (%)	pT1/2/3/4 (%)	pN0/1/2/3 (%)	M1 (%)	CY1 (%)	P1 (%)	Surgical resection (%)	Total gastrectomy (%)	R1/R2 (%)	Median survival (months)	Median survival unresected (months)	Median survival resected (months)	Median survival R0 (months)	1Y-OS (%)	3Y-OSS (%)	5Y-OS (%)	Impact of LP on prognosis after adjustment
Aranha, 1989 [76]	Retrospective cohort	26	–	–	–	–	–	–	50	85	–	6.9	6.6	7.2	–	–	–	–	Not performed
Hamy, 1999 [77]	Retrospective cohort	86	–	5.5(T1 + T2)/4/90.5	–	–	–	–	86	69	–	–	–	12	–	50^d^	40^d^	10^d^	Not performed
Kodera, 2004 [70]	Retrospective cohort	47	–	0/2/72/26	2/13/23/45	–	43	43	83	–	47	14						10	Not performed
Kodera, 2008 [75]	Retrospective cohort	178	–	-/-/-/89	15/23/21/41	–	–	44	84	77	45	13.8	7.8	–	30.2	–	–	–	Not performed
Schauer, 2011 [15]	Retrospective cohort	120	–	0/0/65/35	13/19/18/50	H1: 10%; LYMPH1: 20%	16	33	100	62.5	69	8	–	8	17	–	–	8^d^	Not performed
Endo, 2012 [16]	Retrospective cohort	19	5/5/58/32	0/11/68/21	N+: 89.5%	–	53	46	100	–	–	9^a^	–	9^a^	–	26^d^	21^d^	7^d^	Not performed
Pedrazzani, 2012 [14]	Retrospective cohort	102	–	0/78(T2 + T3)/22	4/37/42/17	–	–	–	59	–	31	5.7	2.8	–	16	68^e^	14^e^	4^e^	Not performed
Jafferbhoy, 2013 [19]	Case series	8	–	–	–	25	–		0	–	–	–	–	–	–	–	–	–	Not performed
Blackham, 2016 [17]	Retrospective cohort	58	III-IV: 90%	2/2/43/53	15/14/28/43	–	–	–	100	88	34	11.6	–	11.6	21.5^a^	–	24^d^	15^d^	No^b^
Thompson, 2016 [78]	Retrospective cohort	54	–	6/12/53/29	24/24/6/41	52	–	–	31	–	35	–	3.6	16.7	–	–	–	–	No^c^

*1Y*-*OS* 1-year overall survival, *3Y*-*OS* 3-year overall survival, *5Y*-*OS* 5-year overall survival

^a^Extracted from the Kaplan-Meier curve

^b^Adjustment by stage and optimal resection status in comparison with non-LP patients

^c^Adjustment by optimal resection status in comparison with non-LP patients

^d^After resection

^e^After R0 resection

In general, scirrhous tumors present a unique biologic behavior, which is believed to be more aggressive than that of other gastric tumors, with a specific tropism for lymph-node and peritoneal involvement [16]. Prognosis of LP patients seem also to be dismal, with frequent presentation with advanced stage disease, poorer disease-free survival, higher rate of peritoneal recurrence, low rate of hepatic metastases, and a median overall survival rate ranging from 5.7 to 13.8 months [14–17, 19, 70, 75–78]. Some authors have also reported a high non-curative resection rate [14, 15, 17, 70, 75, 78], with LP disease often detected in the resection margins [17, 75]. Nevertheless, some of the studies report an equivalent prognosis for LP and non-LP patients when results were adjusted by stage [17], or detected a prognostic advantage for LP patients undergoing R0 resection [14, 15, 17, 75].

Most studies have been conducted on small, non-standardized Western populations [14, 15, 17]. Studies on LP often present mixed populations of curative-intent versus palliative-intent patients, and apply multivariate analysis as adjustment method on small sample sizes. In those analyses, possible confounders and effect modifiers are not always considered, and it is still not clear if LP may represent a real independent prognostic factor or a confounder. As randomization is not easily feasible in this setting, an interesting approach may be the use of propensity-score matching [79] to balance the characteristics between LP and non-LP patients. Waiting for new evidences, the debate is still open. Meanwhile, patients should not be denied treatment on the basis of a preoperative diagnosis of LP when curative-aim surgery is possible.

### Linitis plastica: implications for therapy

As soon as the possibility of a curative treatment for a patient with LP is assessed, other questions arise in regards to the therapeutic management.

For years, ample resection margins (>5 cm) have been advocated to avoid R1 resection in GC patients, and they are considered the current standard for patients with Borrmann III and IV tumors, in accordance with the Japanese Guidelines [80]. This topic, however, is extremely controversial. Indeed, a discrete number of studies seem to have disproved the value of wide resection margins, as long as a R0 resection is obtained, while other studies have even been questioning the role of R0 resection in advanced stages [81, 82]. However, studies focusing on diffuse, SRC, and especially on scirrhous tumors are lacking. Thus, caution is needed when performing limited resections in these subgroups. Given its high accuracy [17, 81], a frozen tissue biopsy should be routinely performed. If a frozen tissue biopsy is not available, a margin of 5 cm currently remains the gold standard. It should also be considered that in scirrhous gastric cancers and LP phenotypes a frozen tissue could be less reliable due to the lack of tumor cellularity.

Recently, much attention has been given to the use of preoperative hyperthermic intraperitoneal chemotherapy (HIPEC) in GC, both for the prevention of peritoneal disease and for its treatment, and a few randomized investigations are ongoing [83]. Given the strong peritoneal tropism of scirrhous and LP tumors, in the near-future, HIPEC could come to the foreground for the routine management of this subgroup of patients.

Neoadjuvant therapy has many theoretical advantages. Among them are the higher rate of treatment compliance in comparison to postoperative therapy, and the possibility of downstaging or downsizing the tumor [84]. As LP tumors often present in an advanced stage, neoadjuvant therapy may be of particular value in improving local control and increasing the rate of potentially curative gastrectomies.

Nevertheless, concerns remain when applying conventional therapeutic agents. The use of radiotherapy as an adjuvant treatment was significantly less effective in diffuse tumors in both the ARTIST trial and the 10-year update of the INT-0116 trial [85, 86]. In vitro testing on the G-DIF gene-expressing subtype demonstrated reduced sensitivity to 5-fluorouracyl and oxaliplatin in comparison to cisplatin [41]. In a 2004 survey, the use of cisplatin as intraperitoneal preoperative lavage in patients with CY+ schirrous tumors gave no survival benefit in comparison to non-operative management [29]. HER-2 activating mutations are rare between the GS, the MSS/EMT, and the mesenchymal metabolic subtypes [42–44], so Trastuzumab is rarely an option. Finally, in a retrospective French study, patients with SRC tumors have been reported to show scarce response to standard perioperative chemotherapeutic regimens and poor survival [87], due to possible progression of the disease during neoadjuvant therapy, and a phase III randomized clinical trial is currently ongoing to assess the role of perioperative versus adjuvant chemotherapy in this subset [88].

At this point, further studies should be conducted to assess chemo- and radiosensitivity to standard regimens in schirrous tumors and LP, and to evaluate if, in this subset of patients, neoadjuvant treatments are more appropriate than upfront gastrectomy plus adjuvant therapy. Similarly, advancements are needed in identifying the efficacy of different HIPEC drugs.

Currently, targeted therapy is considered only for certain GC subtypes [45], and almost all the chemotherapeutic regimens are directed solely against the cancerous cells. Scirrhous tumors, though, have several distinct features that may be strictly related to their aggressive biological behavior. Their desmoplastic stroma may represent both an enhancer of tumor cell growth and invasiveness [53] and a shield against the host’s immune response and against standard chemotherapy [51]. On the one hand, this consideration may be a topic in favor of upfront gastrectomy (with the strategy of removing the “shield,” then treating minimal residual disease); on the other, it should prompt the development of agents targeted against the cancer-stroma interaction factors or against the stromal cells. This strategy may represent a real game-change in the context of a multimodal therapeutic management, especially for patients which are non-responders to conventional therapy. In this regard, interesting preliminary effects have been observed with Tranilast (a mast-cell and fibroblast inhibitor) [89], and TGF-B and FGF7 receptor inhibitors [90, 91] in association with standard chemotherapy. Further advancements in the understanding of tumor-stroma interactions are expected.

## Conclusions

Primary linitis plastica of the stomach is a diffuse-type carcinoma with a scirrhous stroma, which invades the submucosa involving more than 1/3 of the stomach surface. Currently, appropriate management strategies include an accurate diagnostic multi-instrumental assessment and diagnostic laparoscopy in all cases. Curative-aim surgery, when feasible, should be performed. For these patients, the role of neoadjuvant therapy has to be assessed, and the impact of standard chemo and radiotherapy protocols should be further analyzed. Pre- or postoperative HIPEC may represent an alternate strategy, especially given the high rates of peritoneal spread of this tumor. Further advancements are needed in regards to the development of targeted therapies, which will address both cancer cells and their stroma. Future studies on scirrhous tumors and LP should be based on a standardized definition such as the one proposed. Moreover, Western pathologist should introduce in routine examinations the assessment of the stromal reaction, to allow for better patients’ stratification and on par comparison with the Eastern setting.

## Abbreviations

18-FDG: 18-Fludeoxyglucose
CAFs: Cancer-associated fibroblasts
CT: Computed tomography
EUS: Endoscopic ultrasound
GC: Gastric cancer
HIPEC: Hypertermic intraperitoneal chemotherapy
LP: Linitis plastica
MRI: Magnetic resonance imaging
PET: Positron emission tomography
SRC: Signet-ring cell
UGI: Upper gastrointestinal series
WHO: World Health Organization
